# Couette flow of viscoelastic dusty fluid in a rotating frame along with the heat transfer

**DOI:** 10.1038/s41598-020-79795-w

**Published:** 2021-01-12

**Authors:** Muhammad Bilal, Salaha Khan, Farhad Ali, Muhammad Arif, Ilyas Khan, Kottakkaran Sooppy Nisar

**Affiliations:** 1 Department of Mathematics, City University of Science and Information Technology, Peshawar, Khyber Pakhtunkhwa Pakistan; 2 Computational Analysis Research Group, Ton Duc Thang University, Ho Chi Minh City, Vietnam; 3 Faculty of Mathematics and Statistics, Ton Duc Thang University, Ho Chi Minh City, Vietnam; 4 Department of Mathematics, College of Science Al-Zulfi, Majmaah University, Al-Majmaah, 11952 Saudi Arabia; 5 Department of Mathematics, College of Arts and Science, Prince Sattam Bin Abdulaziz University, Wadi Al-Dawaser, 11991 Saudi Arabia

**Keywords:** Applied mathematics, Computational science

## Abstract

Viscoelastic fluid is an advanced fluid which exhibits both elastic and viscous properties. Whereas rotation of viscoelastic fluid is a very complex phenomenon and has an immense amount of applications in engineering and product making industries. Due to various applications in real life researchers are working to understand the rheology of viscoelastic fluids. Viscoelastic dusty fluids are used in gas cooling systems. In nuclear reactors, dusty fluids are used to lower the temperature of the system. Such fluids are also used in centrifugal separators, which separate solid particles from the liquid state, etc. Therefore, in the present study, viscoelastic dusty fluid is analyzed. More precisely free convective Couette flow under the influence of the transversely applied uniform magnetic field in a rotating frame is considered. The subject fluid is driven by the sine oscillations of the upper plate along with the effect of free convection. Due to rotation, the fluid and dust particles have complex velocities which is the sum of primary velocity and secondary velocity. The flow regime is modeled in terms of partial differential equations. To non-dimensionalize the system of governing equations, dimensionless variables have been derived through Buckingham-Pi theorem. The system of partial differential equations is solved through assumed periodic solutions (Poincare-Light Hill Technique). The expressions for skin friction (shear stresses at $y = 0$) and Nusselt number (the rate of heat transfer) are also calculated. Moreover, parametric influence on Nusselt number, and velocity profile of the fluid and the dust particles, is discussed. It is observed that increase in rotation parameter η causes retardation in the velocity of dust particles and the fluid. This is due to the fact that the increase in η enhances the Coriolis forces that are in fact inertial forces, causing a retardation in the velocity of the fluid phase and the dust phase.

## Introduction

Multiphase magnetohydrodynamic (MHD) flows are of great importance due to their extremely useful applications like fluidization, MHD generators, dusty plasma devices (DPDs), use of dust in cooling systems, and nuclear reactors. Multiphase flow can also be observed in nature such as sediment transport in rivers, in which suspended particles are the solid phase whereas water is the liquid phase. Multiphase flow can also be seen in human bodies i.e. blood flow in human bodies, in which plasma is the liquid phase and red blood cells are referred as solid. The most simple case in multiphase flow is the two-phase flow, which could be a combination of liquid–gas flow, solid–gas flow, liquid–liquid flow and liquid–solid flow.

Viscoelastic fluids are those fluids which posses both the characteristics of viscosity and elasticity which are the traits of fluids and solids respectively. Viscoelastic materials are extremely good shock absorber, therefore, they have many applications in industrial and biomedical sciences especially in the blood flow. Blood flow is viscoelastic in nature due to its elastic energy which is stored in the circulatory system and then used to produce heat energy. The remaining energy is utilized in the movement of the body and other functions^1^. Oldroyd^2^ investigated the creeping nature of viscoelastic fluid by modeling the governing equation and studied the influence of non-Newtonian fluid flow by using Oldroyd fluid model. Rajagopal and Bhatnagar^3^ studied the Oldroyd fluid model and found analytical solutions.

Micheal and Miller^4^ studied the dusty gas and its flow generated by the motion of an infinite plate. They utilized the Saffman formulation^41^ by considering two types of motion of the parallel plate i.e. simple harmonic motion and abrupt change in the resting position of the plane which moves with the uniform velocity. Soo^5^ initiated and analyzed the basic theory of multiphase flow. Venkatesh and Kumara^6^ also worked on the unsteady flow of conducting dusty fluids flowing between vibrating plates along a wavy wall. They found that the velocity profile for fluid as well as for dusty particles is parabolic in nature, and tends to become zero for a larger value of time but at the center of the channel the velocities become minimum. Later Ghosh and Sana^7^ analyzed the motion of dusty fluids with spherical dust particles under the effect of magnetic field which is transversely applied to the flow. Many other researchers like Saffman^8^, Vimala^9^, Healy^10^, Venkateshappa et al.^11^, Gupta and Gupta^12^, Ghosh and Ghosh^13^, Attia and Abdeen^14^, Ghosh and Debnath^15^, Gireesha et al.^16^, etc., investigated the theoretical modeling and experimental measurement of particles phase velocity in a multiphase dusty fluid. Ali et al.^17^ studied the generalized two phase flow of blood consisting of magnetic particles. They considered fractional model with the effect of isothermal heating. This study was mainly focused to understand the impact of magnetic field and its uses in human blood. In another paper, Ali et al.^18^ investigated the effect of heat transfer on the two phase blood flow with embedded magnetic particles. They used the newly developed definition of Caputo Fabrizio fractional derivative. Recently Ali et al*.*^19^ studied the fluctuating two-phase flow of viscoelastic dusty fluid between two horizontal inelastic plates. The effect of heat transfer with the influence of magnetic field was taken into account. Unlike the published work in this investigation, they concluded that the flow in boundary layer increases with the greater value of magnetic field. More recently, Ali et al.^20^ analyzed the fluctuating unsteady free convective flow of viscoelastic fluid with embedded dust particles under the impact of MHD. They discussed the variation of velocity of dust phase and the fluid phase in boundary layer and in free stream with the dual behavior of magnetic parameter.

In astrophysics, engineering sciences and biomedical sciences MHD free convective flow is of prime importance. Free convective MHD flows are also used in the fluid engineering science side such as, they are used in MHD generators, for cooling systems, combustion chambers, radiators, adjusting blood flow during surgery, etc. Due to the above mentioned immensely valuable applications researchers analyzed and studied this phenomenon in each and every possible corner such as^21–29^, investigated the MHD free convective flow, which is of key importance in real world and has many industrial applications like many exothermic and endothermic chemical reactions, removal of heat from nuclear reactors, storage of edible goods, etc. Sheikh et al.^30^, studied the generalized form of Casson fluid with chemical reaction and compared the two different fractional operators. Khan et al.^31^, discussed the effect of chemical reaction on MHD free convective flow over a moveable plate submerged in a semi-permeable medium. Sahoo et al.^32^ scrutinized the free convective MHD flow of electrically conducting incompressible viscous fluid past over a semi-permeable plate, with the effect of heat transfer. Chamkha^33^ investigated the unsteady MHD flow of suspension in a conductive fluid flowing in a channel, considering the effect of thermal radiation. Kumar et al*.*^34^ studied micro polar and viscous fluid in a vertical channel. They considered the flow to be fully developed and also considered free convection. They found an increasing behavior of velocity with increasing Grashof to Reynolds number ratio, viscosity ratio and width ratio while a decreasing behavior was noticed with increasing micro polar fluid material parameter. A hydromagnetic dusty flow of an electrically conducting fluid has been studied by Chamkha^35^. He has evaluated the analytical solutions and found that owing the existence of dust in channel, the flow rate of fluid-phase and dust-phase both decreases. In another paper Chamkha^36^ has studied the time dependent flow of electrically conducting dusty-gas between two parallel plates. He has found that decrease in volume flow rate of fluid-phase, volume flow rate of dusty-phase occurs by loading the dust particles in channel. Similarly Chamkha^37^ has also discussed MHD flow in channel with free convection. He has also found various analytical solutions for the velocity profile and temperature profile for difference special cases. Furthermore, the Nusselt number for both walls has been calculated. He concluded that no reverse flow ocurrs when the channel is symmetric.

Rotational flows are of immense importance due to their applications in science, engineering, and in product making industries. The science behind the rotational flow provides the basis and modeling capabilities of many manufactured goods such as jet-engines, Vacuum pumps, centrifugal pumps, etc. In nature, rotational flows can also be seen such as in whirlpools, tropical cyclones, tornadoes, etc.Since the rotational flow is a very complex phenomenon, researchers are trying to understand the science behind it. Therefore Gireesha et al.^38^ analyzed the flow of the boundary layer of a dusty fluid having constant angular velocity. They also considered the effect of time-dependent pressure gradient and Hall current. In this investigation they discussed the effect of Ekman number, magnetic parameter, Hall current parameter and time on the fluid’s velocity as well as on the velocity of dust particle. Manjunatha et al.^39^ investigated the series solutions for a rotating dusty fluid having unsteady flow with free convection and radiation effect. They observed the effect of different parameters such as Hall current parameters, rotation parameter, magnetic parameter on velocity profile as well as on the thermal boundary layer. Dey^40^ analyzed the rotating Jeffrey dusty fluid flow over a non-conducting semi-permeable plate, considering volume fraction and Hall current effect. Nazibuddin et al.^41^ studied an incompressible viscous fluid having transient MHD flow past a horizontal accelerated porous plate in a rotating system with Hall current. They analyzed the effects of velocity profile by varying different parameters such as accelerating parameters, magnetic parameters, and rotational parameters. Kanch and Jana^42^ scrutinized the effect of Hall current on a hydromagnetic unsteady flow past a rotating disk. They concluded that for a large time period the steady-state is attained by inertial oscillations. The frequency of the inertial oscillations initially increases, after reaching the maximum value the frequency decreases by increasing the Hall current parameter. Rajagopal^43^ analyzed the viscoelastic fluid flowing between rotating disks.

Keeping in view the above cited work, up to the best of our knowledge no work has been done on free convective MHD Couette flow of viscoelastic dusty fluid in a rotating frame. As, evident from the aforesaid salient applications of rotatory viscoelastic dusty fluid it is very important to fully understand the rheology of such complex phenomena. Therefore, in the present work free convective MHD Couette flow of a viscoelastic dusty fluid in a rotating frame is considered and the velocity is taken as complex velocity for both the fluid and the dust particles. This complex velocity is basically the combination of primary velocity in *x*-direction while the secondary velocity is in *z*-direction due to the fact that the rotation is considered about *y*-axis.

## Mathematical modeling

**Figure 1:**
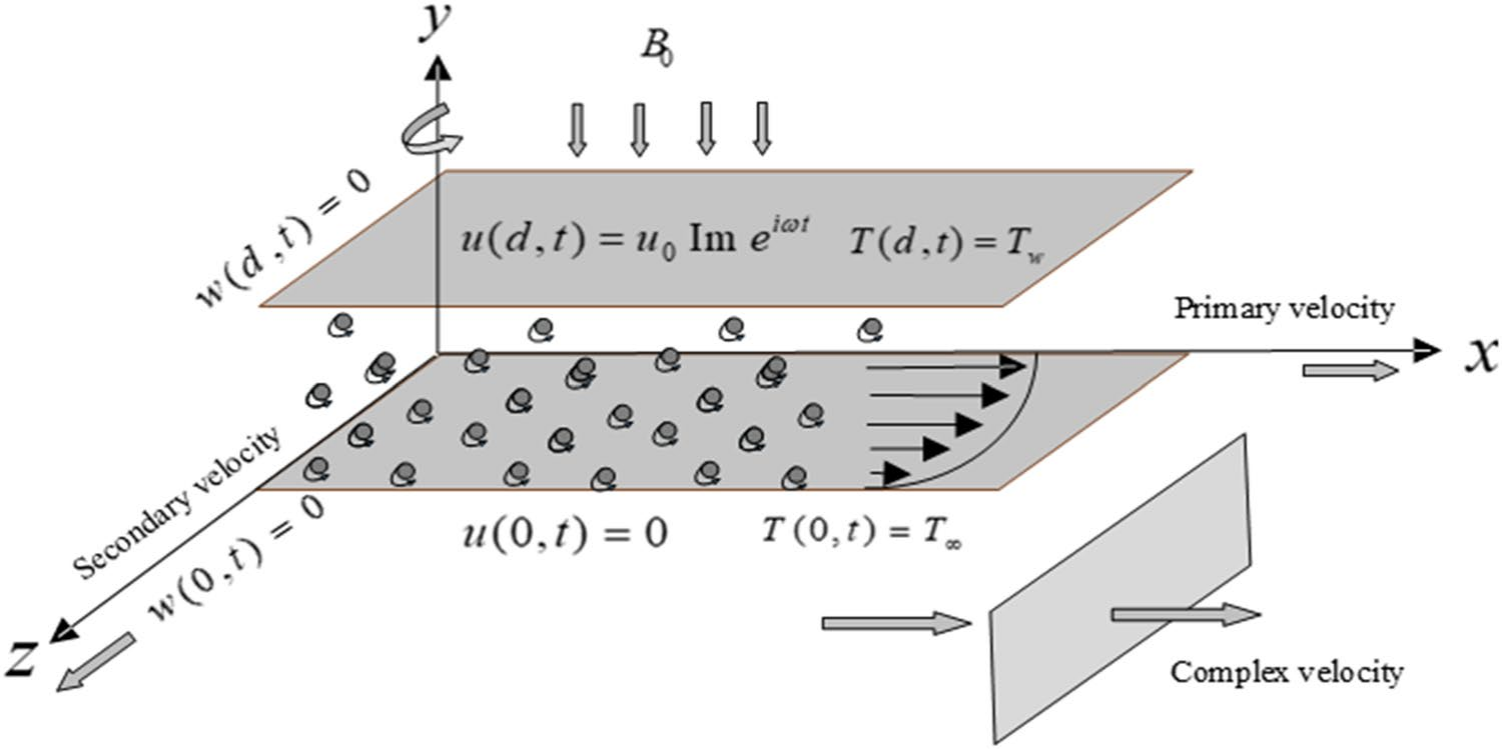
Schematic diagram of the flow.

In this work, an unsteady, incompressible viscoelastic fluid with spherical shaped embedded dust particles in a rotating frame is considered. The infinitely rigid plate is extended in $x$-direction and $z$-direction. It is assumed, that motion of the fluid is along $x$-direction, which cover the $xz$-plane at $y \ge 0$. The subject fluid is electrically conducted and uniform magnetic field $B_{0}$ is applied transversely to the flow direction. The system is considered to be in solid body rotation with a uniform angular velocity $\Omega$. The effect of heat transfer with thermal radiation is also taken into account. Initially, at lower plate the ambient temperature $T_{\infty }$ has been considered. At $t = 0^{ + } ,$ the upper plate starts oscillation, which generates momentum in the fluid, this momentum is enhanced with impact of wall temperature $T_{w}$ as shown in Fig. 1.

The velocity and the temperature fields for the above flow regime are

1 $$\overrightarrow {V} = \left( {u\left( {y,t} \right),\Omega ,w(y,t)} \right),$$

2 $$T = (T(y,t),0,0).\,$$

The basic constitutive equations for the rotating flow of viscoelastic fluid are given as:

3 $$\nabla \cdot \overrightarrow {V} = 0$$

4 $$\rho \left[ {\frac{{d\overrightarrow {V} }}{dt} + 2\left( {\Omega \times \overrightarrow {V} } \right) + \Omega \times \left( {\Omega \times \mathop r\limits^{ \to } } \right)} \right] = div\overrightarrow {{\mathbf{T}}} + \rho \overrightarrow {b} + \overrightarrow {s} ,$$

where T is the Cauchy stress tensor and defined as:

5 $${\mathbf{T}} = - P{\mathbf{I}} + \mu {\mathbf{A}}_{1} + \alpha_{1} {\mathbf{A}}_{2} + \alpha_{2} {\mathbf{A}}_{1}^{2} ,$$

P is the pressure, I is the identity matrix, µ is the viscosity of the fluid, $\alpha_{1} ,\alpha_{2}$ are material parameters, $A_{1} ,A_{2}$ are Revillon Erickson tensors of first and second kind respectively, defined as:

6 $$\begin{aligned} & {\mathbf{A}}_{1} = {\mathbf{L}} + {\mathbf{L}}^{T} , \\ & {\mathbf{A}}_{2} = \frac{{d{\mathbf{A}}_{1} }}{dt} + {\mathbf{A}}_{1} {\mathbf{L}} + {\mathbf{L}}^{T} {\mathbf{A}}_{1} . \\ \end{aligned}$$

$\rho \overrightarrow {b}$ are body forces which includes Lorentz forces and bouncy forces, $\overrightarrow {s}$ is the surface interactive forces that generates due to the interaction between dust particles and fluid particles and defined as:

7 $$\begin{aligned} & \rho \overrightarrow {b} = J \times B + \rho g\beta_{T} (T - T_{\infty } ), \\ & \overrightarrow {s} = K_{0} N_{0} (\overrightarrow {W}_{1} - \overrightarrow {V} ). \\ \end{aligned}$$

Keeping in mind Eq. (1) and using of Eqs. (5–7), Eq. (4) can be written in component form as^44–47^:

8 $$\frac{\partial u(y,t)}{{\partial t}} + 2\Omega w(y,t) = \left( {\upsilon + \frac{{\alpha_{1} }}{\rho }\frac{\partial }{\partial t}} \right)\frac{{\partial^{2} u(y,t)}}{{\partial y^{2} }} + \frac{{K_{0} N_{0} }}{\rho }\left( {w_{1} (y,t) - u(y,t)} \right) - \frac{{\sigma B_{0}^{2} u}}{\rho } + g_{x} \beta_{T} \left( {T - T_{\infty } } \right),$$

9 $$\frac{\partial w(y,t)}{{\partial t}} - 2\Omega u(y,t) = \left( {\upsilon + \frac{{\alpha_{1} }}{\rho }\frac{\partial }{\partial t}} \right)\frac{{\partial^{2} w(y,t)}}{{\partial y^{2} }} + \frac{{K_{0} N_{0} }}{\rho }\left( {w_{2} (y,t) - w(y,t)} \right) - \frac{{\sigma B_{0}^{2} w(y,t)}}{\rho },$$

10 $$m\frac{{\partial w_{1} (y,t)}}{\partial t} + 2\Omega w_{2} (y,t) = K_{0} \left( {u(y,t) - w_{1} (y,t)} \right),$$

11 $$m\frac{{\partial w_{2} (y,t)}}{\partial t} - 2\Omega w_{1} (y,t) = K_{0} \left( {w(y,t) - w_{2} (y,t)} \right),$$

12 $$\frac{\partial T(y,t)}{{\partial t}} = \frac{k}{{\rho c_{p} }}\frac{{\partial^{2} T(y,t)}}{{\partial y^{2} }} - \frac{{\partial q_{r} (y,t)}}{\partial y},$$

where $\frac{{\partial q_{r} }}{\partial y}$ is radiation for optically thin fluid and its value is approximated as^48^

$$- \frac{{\partial q_{r} }}{\partial y} = 4\alpha_{0}^{2} (T - T_{\infty } )$$

The physical initial and boundary conditions are:

13 $$\begin{aligned} & u(y,t) = w(y,t) = 0;\quad at\,t = 0,\,y > 0, \\ & \left. \begin{gathered} u(y,t) = w(y,t) = 0;\quad at\,y = 0, \hfill \\ u(y,t) = u_{0} {\text{Im}} e^{i\omega t} ,w(y,t) = 0;\quad y = d, \hfill \\ \end{gathered} \right\}t > 0 \\ \end{aligned}$$

where $u,w,w_{1} ,w_{2} ,\nu ,\alpha_{1} ,\rho ,K_{0} ,N_{0} ,\sigma ,B_{0} ,g,\beta_{T} ,T,k,$ $\alpha_{0}$ and $C_{p}$ are primary velocity of the fluid, secondary velocity of the fluid, primary velocity of dust particle, primary velocity of dust particle, kinematic viscosity, material parameter, fluid density, stocks resistance coefficient, number density of the dust particles, electrical conductivity, applied magnetic field, applied magnetic field, gravitational acceleration, coefficient of thermal expansion, temperature of the fluid, thermal conductivity, mean radiation coefficient and specific heat capacity of the fluid respectively. Equations (8–11) represents primary and secondary velocity of the fluid and dust particles respectively. The complex velocities for fluid phase and dust phase have obtained from Eqs. (8, 9) and (10, 11), respectively

14 $$\frac{\partial F(y,t)}{{\partial t}} - 2i\Omega F(y,t) = \left( {\upsilon + \frac{{\alpha_{1} }}{\rho }\frac{\partial }{\partial t}} \right)\frac{{\partial^{2} F(y,t)}}{{\partial y^{2} }} + \frac{{K_{0} N_{0} }}{\rho }\left( {W(y,t) - F(y,t)} \right) - \frac{{\sigma B_{0}^{2} F(y,t)}}{\rho } + g\beta_{T} \left( {T - T_{\infty } } \right),$$

15 $$m\frac{\partial W(y,t)}{{\partial t}} - 2i\Omega W(y,t) = K_{0} \left( {F(y,t) - W(y,t)} \right),$$

16 $$\frac{\partial T(y,t)}{{\partial t}} = \frac{k}{{\rho c_{p} }}\frac{{\partial^{2} T(y,t)}}{{\partial y^{2} }} + 4\alpha_{0}^{2} (T - T_{\infty } ),$$

where, $g = g_{x} + g_{y}$ and $g_{y} = 0$, $F(y,t) = u(y,t) + iw(y,t)$, $W(y,t) = w_{1} (y,t) + iw_{2} (y,t)$ are the complex velocities of fluid and dust particles with the transform initial and boundaries conditions:

17 $$\begin{array}{*{20}l} {F(0,t) = 0,} \hfill & {T(0,t) = T_{\infty } } \hfill & {y = 0,\,t > 0,} \hfill \\ {F(d,t) = F_{0} {\text{Im}} e^{i\omega t} ,} \hfill & {T(d,t) = T_{w} ,} \hfill & {y = d,t > 0,} \hfill \\ \end{array}$$

Assume the solution of Eq. (12) obtained through Poincare-Light Hill Technique^49^

18 $$W(y,t) = w_{0} (y)e^{i\omega t} ,$$

Then the complex velocity $W$ of dust particles in terms of complex velocity $F$ of fluid will be

19 $$W(y,t) = \left( {\frac{{K_{0} }}{{mi\omega - 2i\Omega m + K_{0} }}} \right)F(y,t),$$

By incorporating $W$ in Eq. (14) we get:

20 $$\begin{aligned} \frac{\partial F(y,t)}{{\partial t}} - 2i\Omega F(y,t) & = \left( {\upsilon + \frac{{\alpha_{1} }}{\rho }\frac{\partial }{\partial t}} \right)\frac{{\partial^{2} F(y,t)}}{{\partial y^{2} }} + \frac{{K_{0} N_{0} }}{\rho }\left( {\frac{{K_{0} }}{{mi\omega - 2i\Omega m + K_{0} }} - 1} \right)F(y,t) \\ & \quad - \frac{{\sigma B_{0}^{2} F(y,t)}}{\rho } + g\beta_{T} \left( {T - T_{\infty } } \right), \\ \end{aligned}$$

Introducing the dimensionless variables derived by using Buckingham-Pi theorem;

21 $$F^{*} = \frac{F}{{F_{0} }},\quad y* = \frac{y}{d},\quad t* = \frac{{F_{0} t}}{d},\quad \theta = \frac{{T - T_{\infty } }}{{T_{w} - T_{\infty } }},\quad \tau^{*} = \frac{{\tau d^{2} }}{\mu \upsilon },$$

using Eq. (21) , Eq. (16), Eq. (17) and Eq. (20) become dimensionless of the form:

22 $${\text{Re}} \frac{\partial F(y,t)}{{\partial t}} - 2i\eta F(y,t) = \frac{{\partial^{2} F(y,t)}}{{\partial y^{2} }} + \alpha \frac{{\partial^{3} F(y,t)}}{{\partial t\partial y^{2} }} + \left( {K_{2} - K_{1} } \right)F(y,t) - MF(y,t) + Gr\theta (y,t),$$

23 $$Pe\frac{\partial \theta (y,t)}{{\partial t}} = \frac{{\partial^{2} \theta (y,t)}}{{\partial y^{2} }} + N^{2} \theta (y,t);$$

24 $$\begin{array}{*{20}l} {F(0,t) = 0,} \hfill & {\theta (0,t) = 0,} \hfill \\ {F(1,t) = {\text{Im}} e^{i\omega t} ,} \hfill & {\theta (1,t) = 1,} \hfill \\ \end{array}$$

where

25 $$\begin{aligned} & {\text{Re}} = \frac{{F_{0} d}}{\upsilon },\quad \alpha = \frac{{\alpha_{1} F_{0} }}{\rho \upsilon d},\quad K_{1} = \frac{{K_{0} N_{0} d^{2} }}{\rho \upsilon },\quad K_{2} = \frac{{K_{0}^{2} N_{0} d^{2} }}{{\rho \upsilon (mi\omega - 2i\Omega m + K_{0} )}}, \\ & M = \frac{{\sigma B_{0}^{2} d^{2} }}{\rho \upsilon },\quad Gr = \frac{{g\beta_{T} d^{2} (T_{w} - T_{\infty } )}}{{\upsilon F_{0} }},\quad Pe = \frac{{\rho c_{p} F_{0} d}}{k},\quad \eta = \frac{{\Omega d^{2} }}{\upsilon },\quad N^{2} = \frac{{4\alpha_{0}^{2} d^{2} }}{k} \\ \end{aligned}$$

While ${\text{Re}} ,\alpha ,K_{1} ,K_{2} ,M,Gr,Pe,N^{2}$ and $\eta$ represents the dimensionless Reynolds number, second grade parameter, Dusty parameters, magnetic parameter, Grashof number, Peclet number, radiation parameter and dimensionless rotational parameter respectively.

(Note: To avoid complexity * has been dropped).

Consider the following assume periodic solutions for energy equation obtained through Poincare-Light Hill Technique^49^

26 $$\,\,\,\theta (y,t) = \theta_{0} (y) + \varepsilon \theta_{1} (y)e^{i\omega t} + O(\varepsilon^{2} ).$$

By solving energy equation with the help of above assumed periodic solution and ignoring higher order of $\varepsilon$ we get;

27 $$\theta_{0} (y) = \frac{{{\text{Sin}} (Ny)}}{{{\text{Sin}} N}};\,\theta_{1} (y) = 0.$$

Using the values of $\,\,\,\theta_{0} (y)$ and $\,\,\,\theta_{1} (y)$ in Eq. (26), then the final solution of energy equation will be:

28 $$\theta (y,t) = \frac{{{\text{Sin}} (Ny)}}{{{\text{Sin}} N}}$$

Now by coupling energy in momentum equation and assuming the periodic solution obtained through Poincare-Light Hill Technique^49^

29 $$\,\,\,F(y,t) = F_{1} (y) + \varepsilon F_{2} (y)e^{i\omega t} + O(\varepsilon^{2} ).$$

Incorporating the above assume solution in Eq. (22), and separating the harmonic and non-harmonic parts, the following values for $F_{1} (y)$ and $F_{2} (y)$ are obtained

30 $$F_{1} (y) = A\left\{ {\frac{{{\text{Sin}} (Ny)}}{{{\text{Sin}} (N)}} - \frac{{{\text{Sinh}} \left( {\sqrt {M_{1} } y} \right)}}{{{\text{Sinh}} \left( {\sqrt {M_{1} } } \right)}}} \right\},\quad F_{2} (y) = \frac{{{\text{Sinh}} \left( {\sqrt {M_{2} } y} \right)}}{{\varepsilon {\text{Sinh}} \left( {\sqrt {M_{2} } } \right)}},$$

31 $$F(y,t) = A\left\{ {\frac{{{\text{Sin}} (Ny)}}{{{\text{Sin}} (N)}} - \frac{{{\text{Sinh}} \left( {\sqrt {M_{1} } y} \right)}}{{{\text{Sinh}} \left( {\sqrt {M_{1} } } \right)}}} \right\} + \left\{ {\frac{{{\text{Sinh}} \left( {\sqrt {M_{2} } y} \right)}}{{{\text{Sinh}} \left( {\pi \sqrt {M_{2} } } \right)}}} \right\}e^{i\omega t} .$$

where

$$A = \frac{Gr}{{N^{2} + M_{1} }},\quad M_{1} = K_{2} - K_{1} - 2i\eta ,\quad M_{2} = \frac{{K_{2} - K_{1} + M + {\text{Re}} i\omega t - 2i\eta }}{1 + \alpha i\omega }.$$

## Special case

By considering $\eta \to 0$ and $Gr \to 0$ the general solution which reduces Eq. (31) to the following form:

32 $$u(y,t) = e^{i\omega t} \left( {\frac{{\sinh \sqrt {M_{4} } y}}{{\sinh \sqrt {M_{4} } }}} \right)$$

which represents Couette flow of electrically conducting viscoelastic dusty fluid.

## Nusselt number

Nusselt number is a dimensionless number which is the ratio of convective to conductive heat transfer at a boundary in a fluid. The dimensional form of the Nusselt number is given as:

33 $$Nu = \left. {\frac{d}{{(T_{w} - T_{\infty } )}}\frac{\partial T}{{\partial y}}} \right|_{y = 0} .$$

The dimensionless Nusselt number is presented by

34 $$Nu = \left. {\frac{\partial \theta }{{\partial y}}} \right|_{y = 0} = \frac{N}{{{\text{Sin}} [N]}},$$

## Skin friction

The Drag force which is generated by the friction of fluid against the lower plate’s surface at $y = 0$ that is moving through is called skin friction. As the subject fluid is non-Newtonian viscoelastic, so the expression for the skin friction is given by

35 $$\tau = \left( {\mu + \alpha_{1} \frac{\partial }{\partial t}} \right)\frac{\partial F}{{\partial y}}.$$

Using Eq. (21) in the above equation the following dimensionless form for skin friction is obtained

36 $$\tau = {\text{Re}} \frac{\partial F}{{\partial y}} + \alpha \frac{{\partial^{2} F}}{\partial t\partial y}.$$

Incorporating Eq. (31) in Eq. (34) the following expression for skin friction has been obtained

37 $$\tau = \frac{{ - A{\text{Re}} \sqrt {M_{1} } }}{{{\text{Sinh}} \sqrt {M_{1} } }} + \frac{{e^{i\omega t} ({\text{Re}} + i\omega )\sqrt {M_{2} } }}{{{\text{Sinh}} \sqrt {M_{2} } }}.$$

## Discussion and graphical results

In this article the effect of various physical dimensionless parameters on velocity profile of the base fluid as well as the velocity profile of dust particles has been discussed. The changes in skin friction and Nusselt number with these physical parameters have also been analyzed.

**Figure 2:**
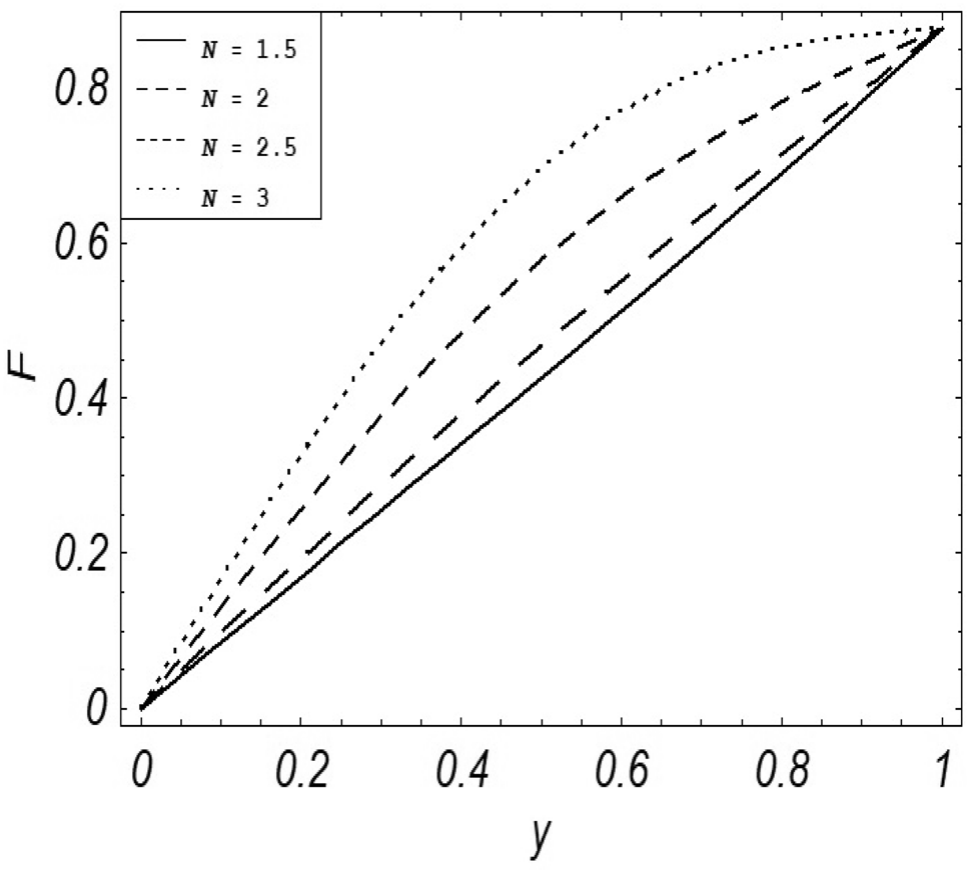
Evolution of $F$ with various values of $N$ if $$\begin{aligned} t = 1;\;Gr = 2.5;\;K_{1} = 0.5;\;\eta = 2;\;M = 0.2; \Omega = 0.5;\;{\text{Re}} = 1;\;\alpha = 0.1;\;\omega = 0.5;\;\varepsilon = 0.001 \\ \end{aligned}$$.

**Figure 3:**
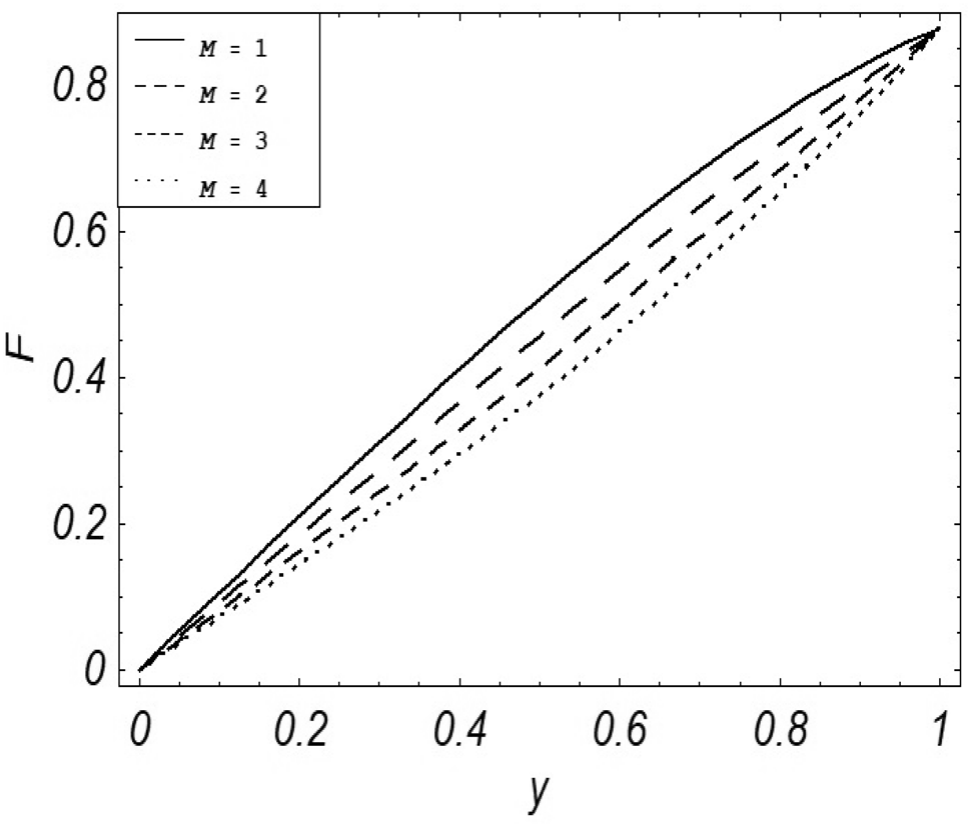
Evolution of $F$ with various values of $M$ if $$\begin{aligned} t = 1;\;Gr = 2.5;\;N = 0.7;\;K = 0.5;\;\eta = 2\;\Omega = 0.5; {\text{Re}} = 1;\;\alpha = 0.1;\;\omega = 0.5;\;\varepsilon = 0.001 \\ \end{aligned}$$.

**Figure 4:**
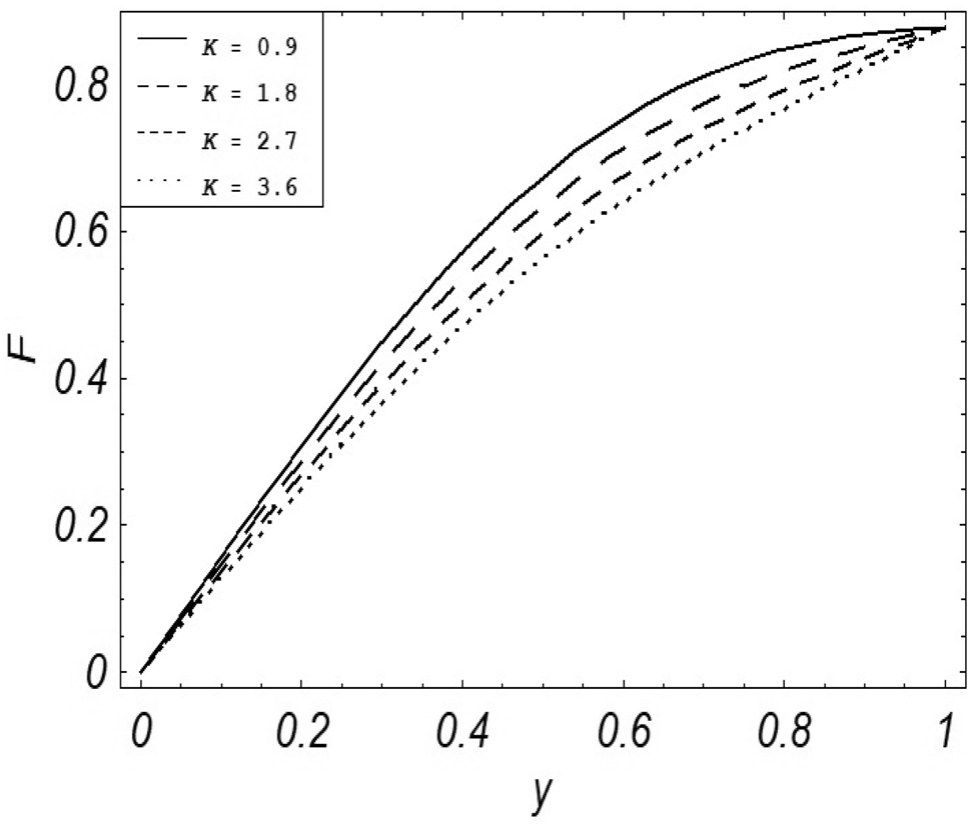
Evolution of $F$ with various values of $K$ if $$\begin{aligned} t = 1;\;Gr = 2.5;\;N = 0.7;\;\eta = 2;\;Gr = 2.5 \Omega = 0.5;\;{\text{Re}} = 1;\;\alpha = 0.1;\;\omega = 0.5;\;\varepsilon = 0.001 \\ \end{aligned}$$.

**Figure 5:**
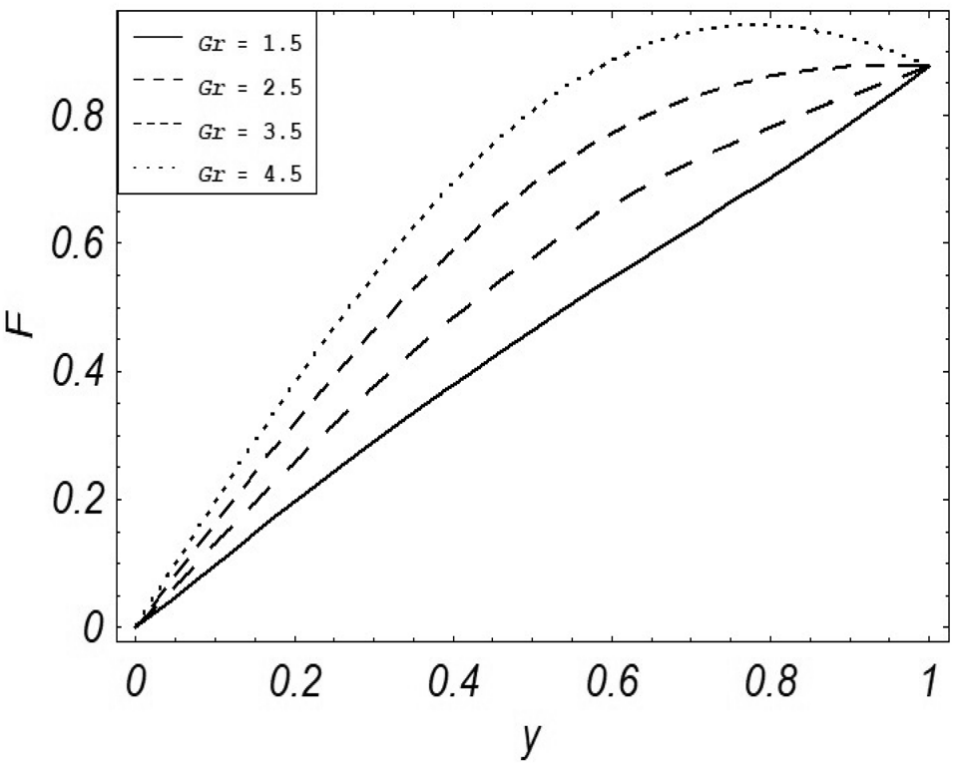
Evolution of $F$ with various values of $Gr$ if $$\begin{aligned} t = 1;\;N = 0.7;\;K = 0.5;\;\eta = 2;\;N = 1 \Omega = 0.5;\;{\text{Re}} = 1;\;\alpha = 0.1;\;\omega = 0.5;\;\varepsilon = 0.001 \\ \end{aligned}$$.

**Figure 6:**
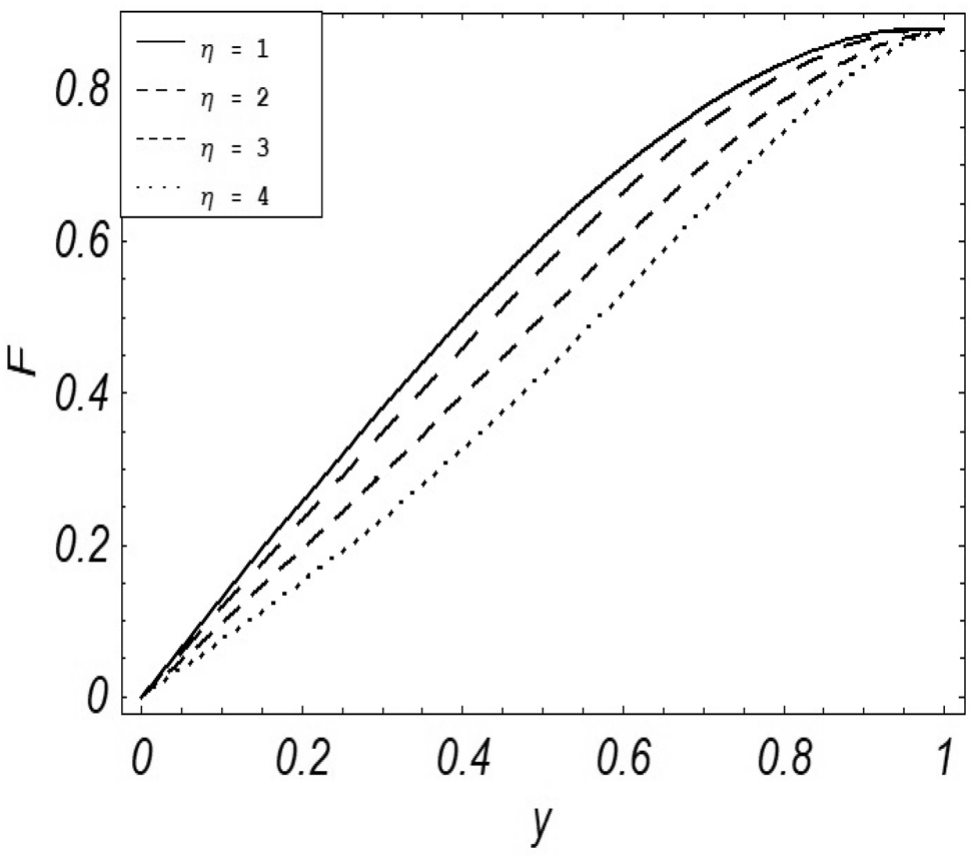
Evolution of $F$ with various values of $\eta$ if $$\begin{aligned} t = 1;\;Gr = 2.5;\;K_{1} = 0.5;\;N = 1;\;M = 0.2; \Omega = 0.5;\;{\text{Re}} = 1;\;\alpha = 0.1;\;\omega = 0.5;\;\varepsilon = 0.001 \\ \end{aligned}$$.

**Figure 7:**
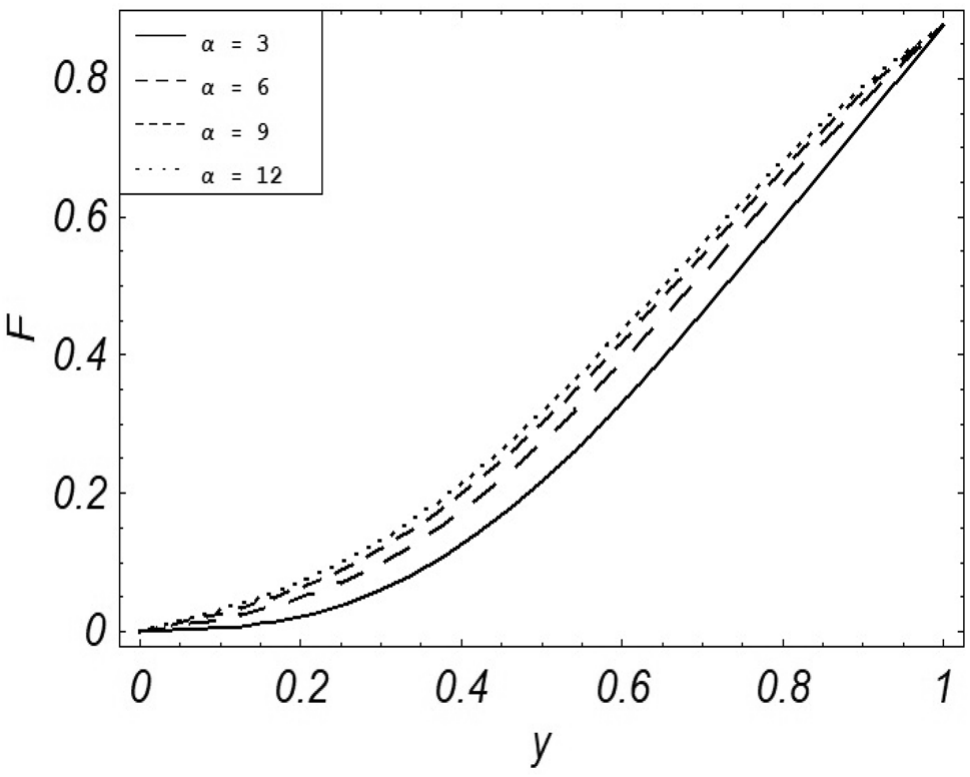
Evolution of $F$ with various values of $\alpha$ if $$\begin{aligned} t = 1;\;Gr = 2.5;\;N = 0.7;\;K = 0.5;\;\eta = 2\;\Omega = 0.5; {\text{Re}} = 1;\;M = 0.1;\;\omega = 0.5;\;\varepsilon = 0.001 \\ \end{aligned}$$.

**Figure 8:**
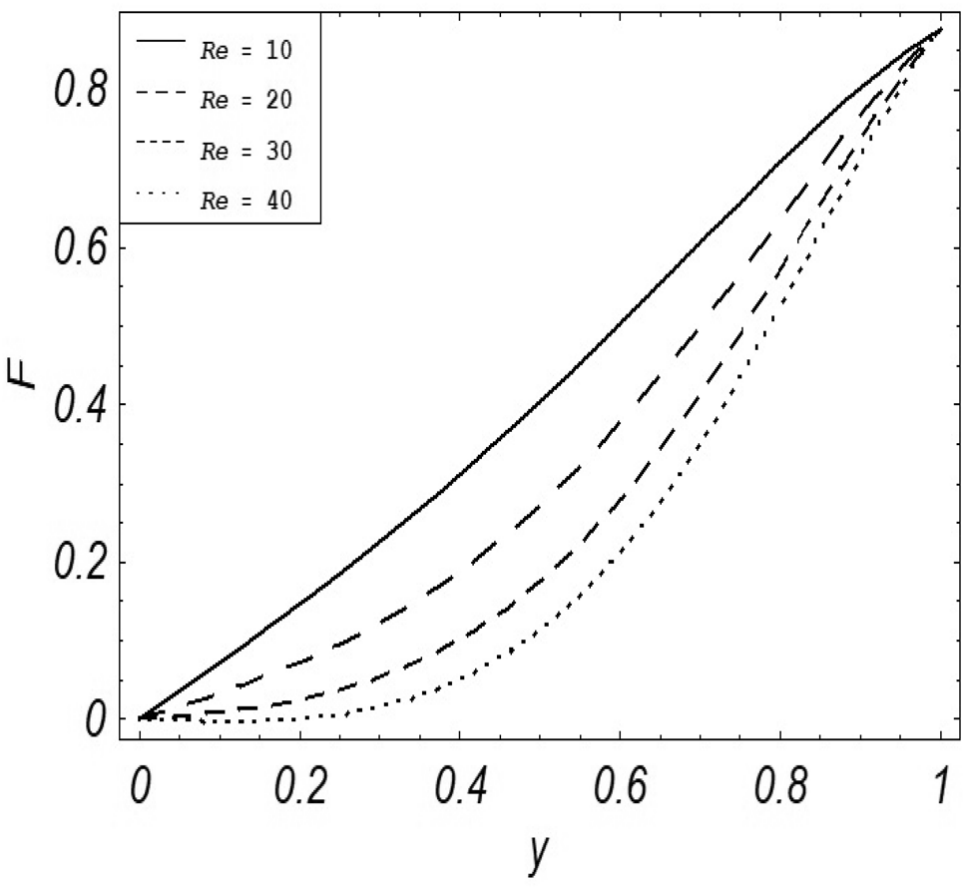
Evolution of $F$ with various values of ${\text{Re}}$ if $$\begin{aligned} t = 1;\;Gr = 2.5;\;N = 0.7;\;\eta = 2;\;Gr = 2.5 \Omega = 0.5;\;K = 1;\;\alpha = 0.1;\;\omega = 0.5;\;\varepsilon = 0.001 \\ \end{aligned}$$.

**Figure 9:**
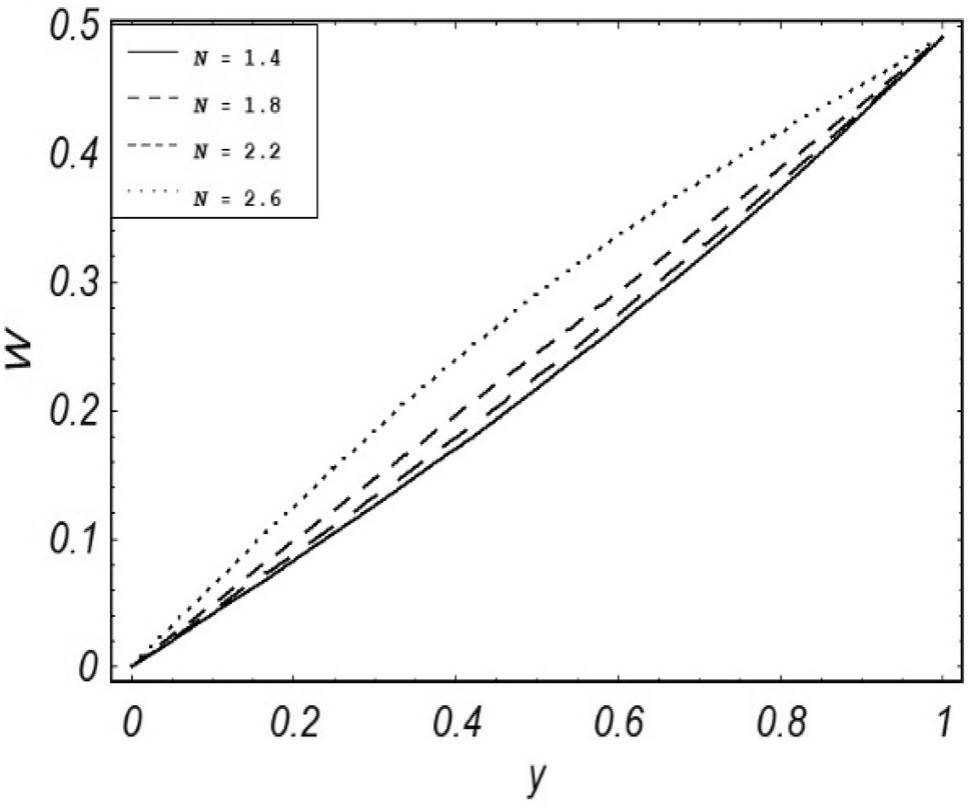
Evolution of $W$ with various values of $N$ if $$\begin{aligned} t = 1;\;Gr = 0.7;\;K = 0.5;\;\eta = 2;\;\Omega = 0.5; {\text{Re}} = 1;\;\alpha = 0.1;\;\omega = 0.5;\;\varepsilon = 0.001;\;m = 0.5 \\ \end{aligned}$$.

**Figure 10:**
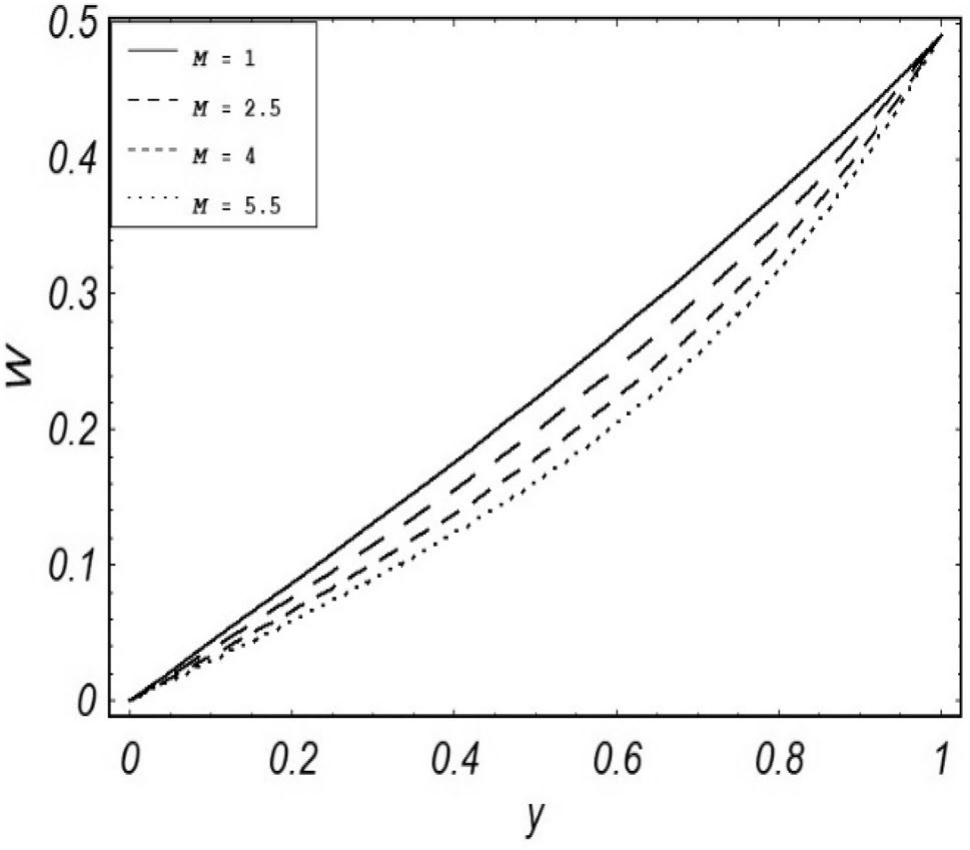
Evolution of $W$ with various values of $M$ if $$\begin{gathered} \,t = 1;\,Gr = 2.5;\,\,K_{1} = 0.5;N = 1;\eta = 0.2;\,\,m = 0.5; \hfill \,\Omega = 0.5\,;\,{\text{Re}} = 1;\,\alpha = 0.1;\,\omega = 0.5;\,\varepsilon = 0.001 \hfill \\ \end{gathered}$$.

**Figure 11:**
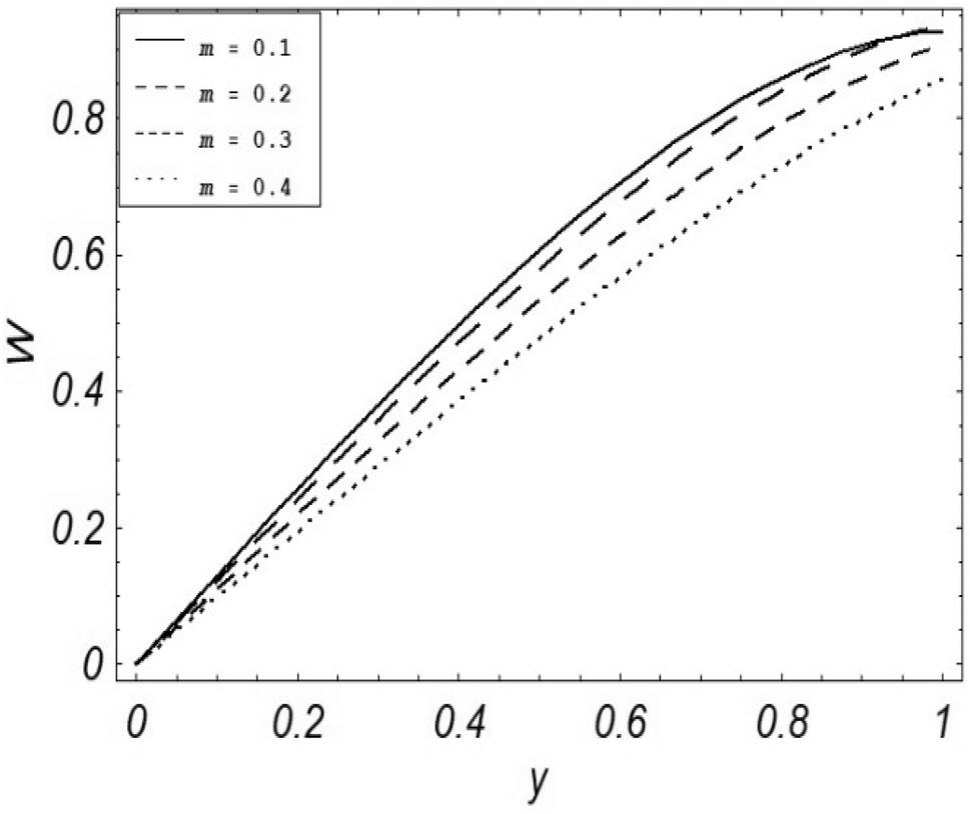
Evolution of $W$ with various values of $m$ if $$\begin{aligned} t = 1;\;Gr = 2.5;\;N = 0.7;\;K = 0.5;\;\eta = 2\;\Omega = 0.5; {\text{Re}} = 1;\;M = 0.1;\;\omega = 0.5;\;\varepsilon = 0.001;\;\alpha = 0.5 \\ \end{aligned}$$.

**Figure 12:**
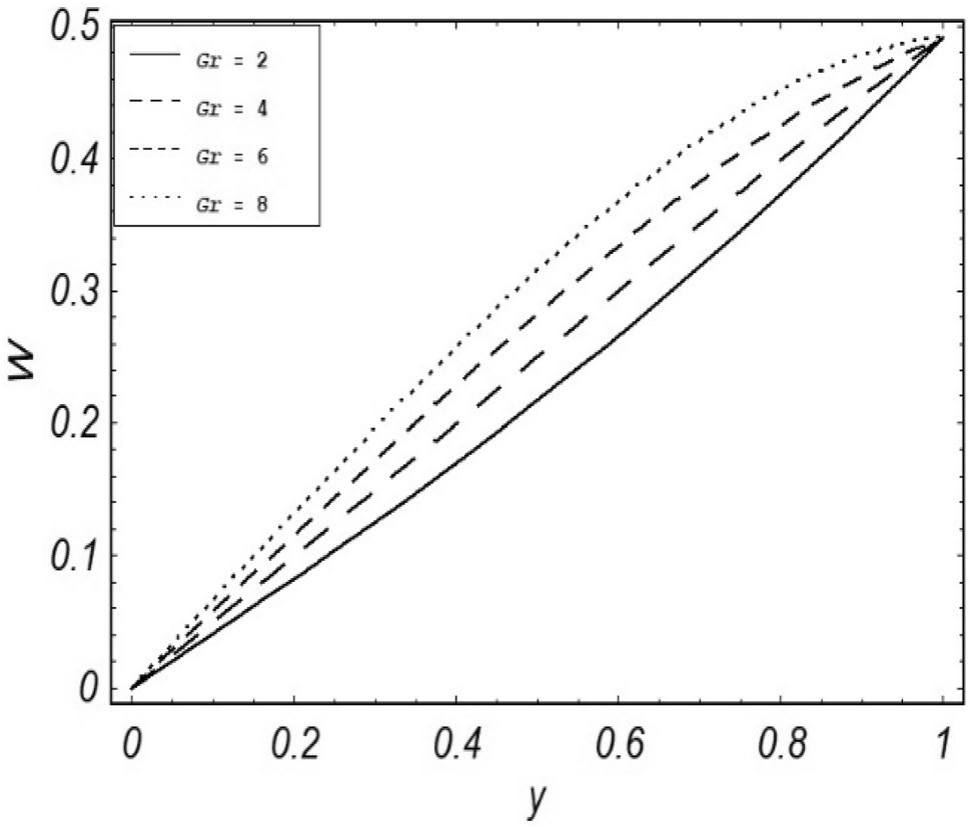
Evolution of $W$ with various values of $Gr$ if $$\begin{aligned} t = 1;\;M = 2.5;\;K_{1} = 0.5;\;N = 1;\;\eta = 0.2;\;m = 0.5; \Omega = 0.5;\;{\text{Re}} = 1;\;\alpha = 0.1;\;\omega = 0.5;\;\varepsilon = 0.001 \\ \end{aligned}$$.

**Figure 13:**
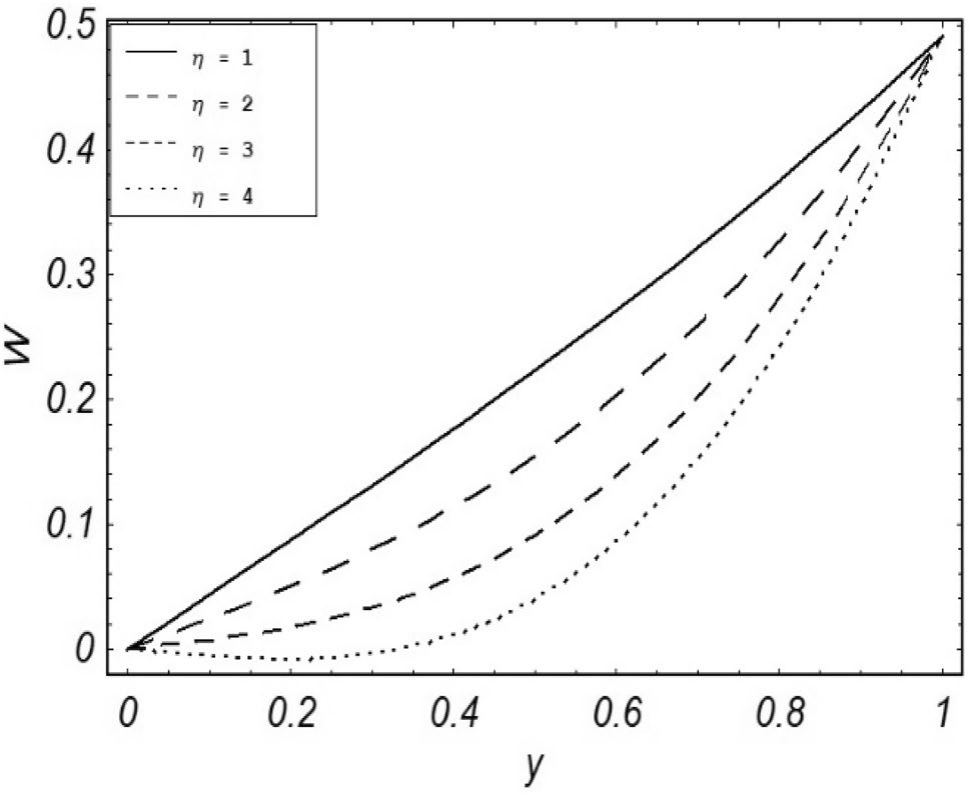
Evolution of $W$ with various values of $\eta$ if $$\begin{aligned} t = 1;\;M = 2.5;\;K_{1} = 0.5;\;N = 1;\;Gr = 0.2;\;m = 0.5; \Omega = 0.5;\;{\text{Re}} = 1;\;\alpha = 0.1;\;\omega = 0.5;\;\varepsilon = 0.001 \\ \end{aligned}$$.

**Figure 14:**
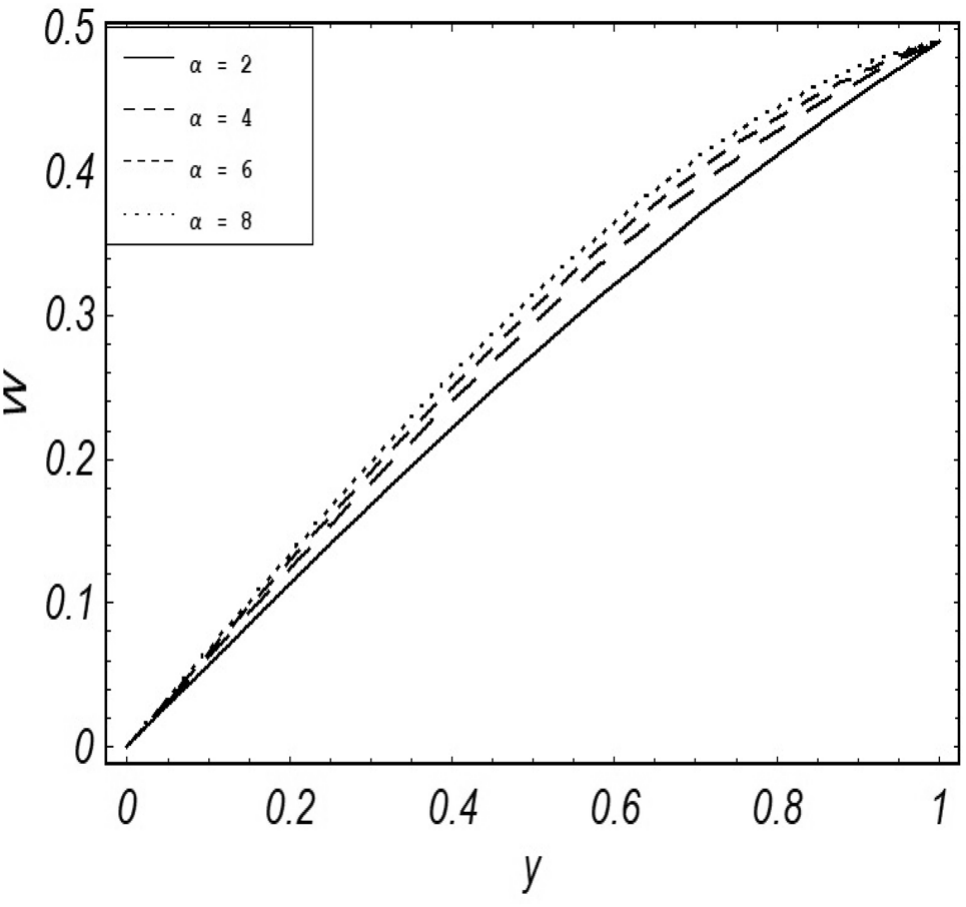
Evolution of $W$ with various values of $\alpha$ if $$\begin{aligned} t = 1;\;Gr = 2.5;\;K = 0.5;\;N = 1;\;\eta = 0.2;\;m = 0.5; \Omega = 0.5;\;{\text{Re}} = 1;\;M = 0.5;\;\omega = 0.5;\;\varepsilon = 0.001 \\ \end{aligned}$$.

**Figure 15:**
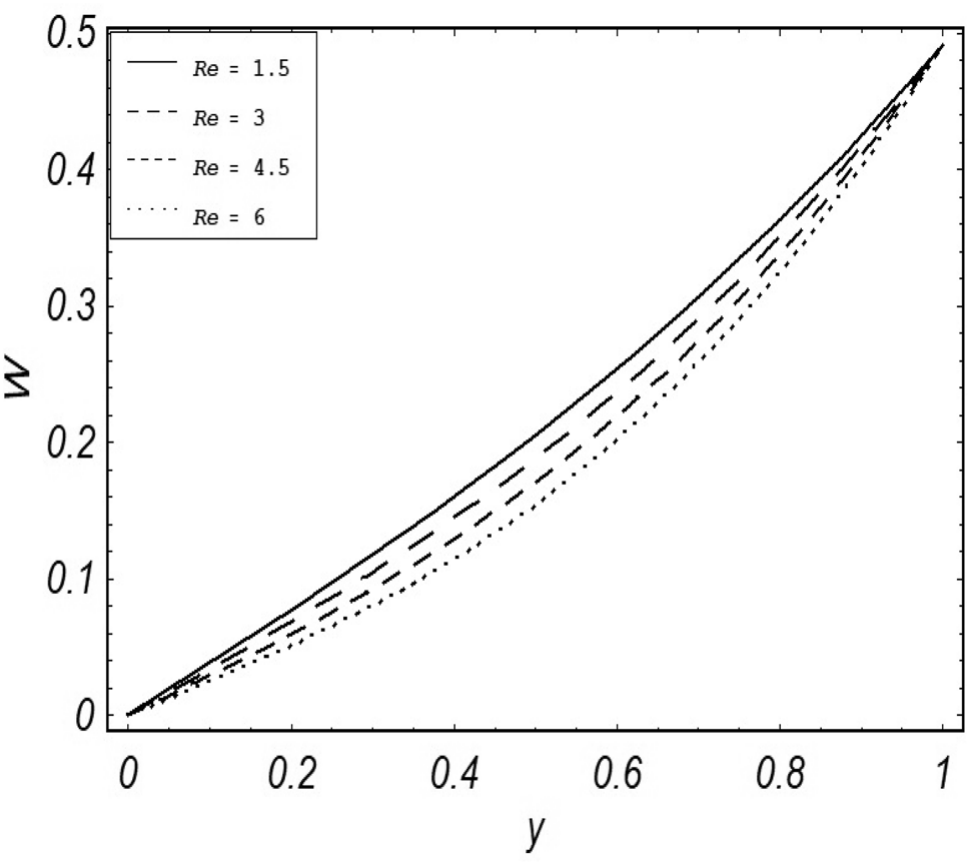
Evolution of $W$ with various values of ${\text{Re}}$ if $$\begin{aligned} t = 1;\;Gr = 2.5;\;N = 0.7;\;K = 0.5;\;\eta = 2\;\Omega = 0.5; m = 1;\;M = 0.1;\;\omega = 0.5;\;\varepsilon = 0.001;\;\alpha = 0.5 \\ \end{aligned}$$.

**Figure 16:**
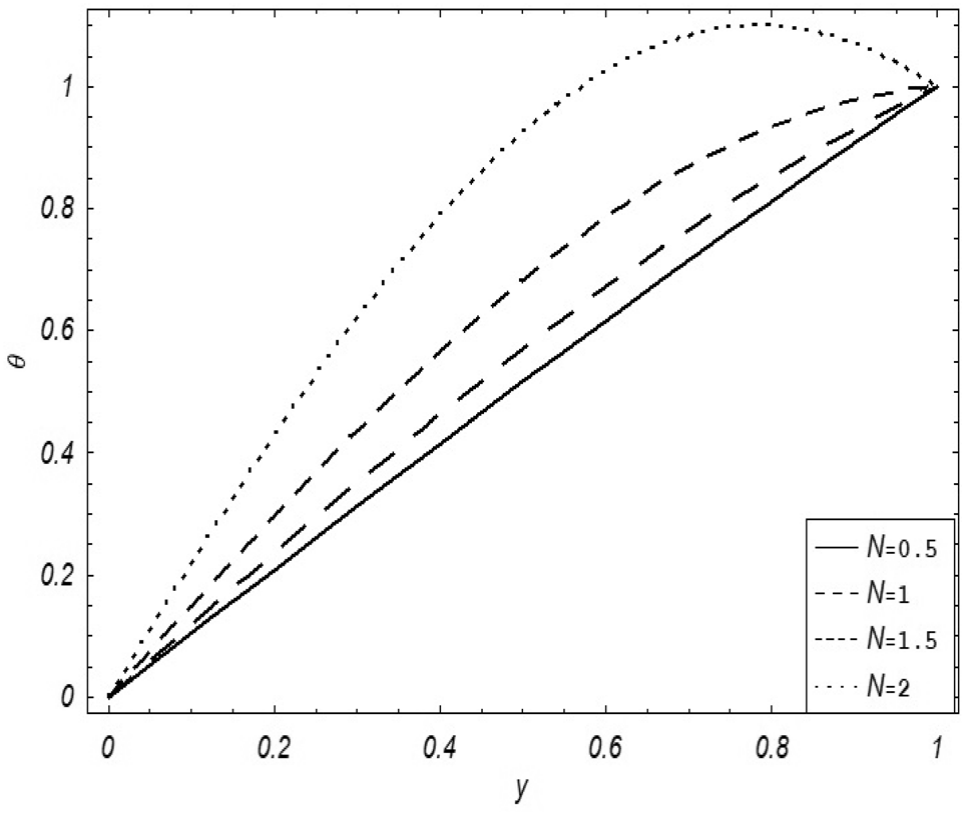
Variation of temperature with $N$.

Figures 2, 3, 4, 5, 6, 7 and 8 represent the behavioral changes in velocity profile of the fluid by varying different physical parameters like Magnetic parameter $M{\kern 1pt}$, Reynolds number Re, Second-grade parameter ∝, Radiation parameter *N,* Dusty fluid parameter *K,* Rotational parameter η, Grashoff Number *Gr*. While Figs. 9, 10, 11, 12, 13, 14 and 15 are presenting the behavioral changes of the dust particle’s velocity with the variation of all the above mentioned physical parameters. The variation of the velocity profile of dust particles is also analyzed by varying mass *m* of the dust particle. In Fig. 16 the effect on temperature with the radiation parameter *N* is shown.

It is noticed that by increasing *M* considerably retards the velocity of the fluid as well as the dust particles. By escalating *M*, Lorentz forces are enhanced which enlarges the resistive forces present inside of a fluid, due to the increase of these resistive forces the velocity of the fluid phase and dusty phase retards. Gr is the ratio of buoyancy forces to viscous forces hence increasing the values of Gr increases the buoyancy forces and decreases the viscous forces and as a result accelerates the velocities of the fluid and the dust particles. We have considered laminar flow of a viscoelastic dusty fluid. In laminar flow viscous forces are significant. Re is the ratio of inertial forces to viscous forces, therefore by increasing the Reynolds number inertial forces are increased which retards the flow. Rotational parameter ${\upeta = \Omega }d^{2} /\upupsilon$ causes retardation in the velocities of dust particles and the fluid due to the fact that the increase in η enhances the Coriolis forces that are in fact inertial forces. Moreover, increasing η decreases υ where $\upsilon = \mu /\rho$ hence with the decrease of viscous forces inertial forces are enhanced, making inertial forces strong enough to retard the velocities of the fluid phase and the dust phase. It is evident from Figs. 2 and 9 that the increase in radiation parameter *N* escalates the velocity of both the fluid phase and the dust phase. This is due to the fact that *N* has a direct relation with temperature which increases the internal kinetic energy of the subject fluid. Since in this article the dust particles are assumed to be in a spherical shape and are homogeneously distributed throughout the subject fluid. According to the Stokes Law drag force $F_{d} = 6\pi \mu r$ hence by increasing concentration of dust particles K, will increase this drag force which in turn increases the viscosity of the fluid rendering to the decrease in the velocity of the fluid phase and the dust phase. Figure 11 shows retarding behavior of the velocity of the dust particles with the increasing value of mass $m$ because of the fact that force and mass have a direct relation, so by increasing the mass $m$ will increase the drag force which increases the viscosity rendering a retarding effect on the velocity of the dust particles. Figure 16 shows the relation between radiation parameter *N* and temperature, due to the fact that kinetic energy is increased due to an increase in *N* which enhances the temperature of the fluid ("Supplememtary Information").

The effect of embedded parameters like Magnetic parameter $M{\kern 1pt}$, Reynolds number Re, Second grade parameter ∝, Radiation parameter *N,* Dusty fluid parameter *K,* Rotational parameter η , Grashoff Number *Gr* on the skin friction is given in Tables 1, 2, 3, 4, 5, 6 and 7. The bold values in each of the tables show the variation in that specific parameter which has shown in that column. It is noticed that by increasing *M* , Re, K, η increases the $Cf$ due to the fact discussed earlier that these parameters noticeably increases the inertial forces hence causing an increase in the numerical value of $Cf$ While a decreasing behavior of $Cf$ is observed with the increase of Gr, ∝, *N*, due to the fact that these parameters decreases viscous forces which decreases the $Cf$ Table 8 represents the variation of Nusselt (Nu) with increasing value of radiation parameter *N*. The radiation parameter *N* depicts a direct relation with the Nusselt number that is by increasing *N* an increase in Nusselt number has been observed.

**Table 1:** Influence of radiation parameter $N$ on skin friction.

$\eta$	$t$	$\omega$	$K$	$N$	$M$	$\alpha$	${\text{Re}}$	$Gr$	$C \cdot f$
0.5	1	0.5π	0.5	**1.5**	0.2	0.1	5	0.5	1.3149
0.5	1	0.5π	0.5	**2**	0.2	0.1	5	0.5	1.2595
0.5	1	0.5π	0.5	**2.5**	0.2	0.1	5	0.5	1.1736

**Table 2:** Influence of magnetic parameter $M_{{{\text{on}}}}$ skin friction.

$\eta$	$t$	$\omega$	$C.f$	$N$	$M$	$\alpha$	${\text{Re}}$	$Gr$	$C \cdot f$
0.5	1	0.5π	0.2	0.5	**1**	3	0.5	10	0.60365
0.5	1	0.5π	0.2	0.5	**2**	3	0.5	10	0.65838
0.5	1	0.5π	0.2	0.5	**3**	3	0.5	10	0.67944

**Table 3:** Influence of dusty fluid parameter $C.f$ on skin friction.

$\eta$	$t$	$\omega$	$C.f$	$N$	$M$	$\alpha$	${\text{Re}}$	$Gr$	$C \cdot f$
0.5	1	0.5π	**0.9**	0.5	10	3	0.5	10	0.60365
0.5	1	0.5π	**1.8**	0.5	10	3	0.5	10	0.60956
0.5	1	0.5π	**2.7**	0.5	10	3	0.5	10	0.61655

**Table 4:** Influence of Grashof number $Gr$ on skin friction.

$\eta$	$t$	$\omega$	$C.f$	$N$	$M$	$\alpha$	${\text{Re}}$	$Gr$	$C \cdot f$
1	1	0.5π	0.5	0.1	0.2	0.1	5	**1.5**	1.3149
1	1	0.5π	0.5	0.1	0.2	0.1	5	**2.5**	0.6751
1	1	0.5π	0.5	0.1	0.2	0.1	5	**3.5**	0.00467

**Table 5:** Influence of rotational parameter $\eta$ on skin friction.

$\eta$	$t$	$\omega$	$C.f$	$N$	$M$	$\alpha$	${\text{Re}}$	$Gr$	$C \cdot f$
1	1	0.5π	0.2	0.5	100	3	0.5	10	0.60365
2	1	0.5π	0.5	0.5	100	3	0.5	10	0.610997
3	1	0.5π	0.5	0.5	100	3	0.5	10	0.624351

**Table 6:** Influence of second grade parameter $\alpha$ on skin friction.

$\eta$	$t$	$\omega$	$C.f$	$N$	$M$	$\alpha$	${\text{Re}}$	$Gr$	$C \cdot f$
0.5	1	0.5π	0.2	0.5	100	**3**	0.5	10	0.60365
0.5	1	0.5π	0.2	0.5	100	**6**	0.5	10	0.529004
0.5	1	0.5π	0.2	0.5	100	**9**	0.5	10	0.31335

**Table 7:** Influence of Reynolds number ${\text{Re}}$ on skin friction.

$\eta$	$t$	$\omega$	$C.f$	$N$	$M$	$\alpha$	${\text{Re}}$	$Gr$	$C \cdot f$
1	1	0.5π	0.5	0.1	100	0.1	**10**	0.5	0.62331
1	1	0.5π	0.5	0.1	100	0.1	**20**	0.5	1.3149
1	1	0.5π	0.5	0.1	100	0.1	**30**	0.5	1.92353

**Table 8:** Variation in Nusselt number with radiation parameter $N$.

Radiation parameter $N$	0.3	0.5	0.7	0.9
Nusselt number $Nu$	1.01516	1.04291	1.08659	1.14895

## Conclusion

The present article deals with the rotational viscoelastic dusty fluid under the impact of magnetic field $B_{o}$ which is being applied transversely to the fluid flow with free convection and radiation. A detailed parametric influential analysis has been done on the complete system of the fluid. Keeping in view the graphical results and discussions we can summarize the whole analysis in the following key-points;

- $N,Gr,$ and $\alpha$ shows a direct relationship with the velocity of the fluid phase and the dust phase.
- $M,K,\eta$ and ${\text{Re}}$ shows an inverse relation with the velocity of fluid phase and the dust phase.
- Enlarging mass $m$ of the dust particles contributes to the decrease in the velocity of the dust phase.
- Skin friction shows a decreasing behavior with the escalating values of $N,Gr$ and $\alpha$.
- While the opposite behaviour is observed in the case of $M,K,\eta$ and ${\text{Re}}$ i.e. an increase in skin friction is noticed with the greater values of $M,K,\eta$ and ${\text{Re}}$.
- The temperature profile of the fluid escalates with the greater value of radiation parameter $N$.
- A direct relation between the Nusselt number and radiation parameter $N$ has been noticed.

## Supplementary Information

Supplementary Information.
