# Supplementary material for: Couette flow of viscoelastic dusty fluid in a rotating frame along with the heat transfer
Source: Sci Rep. 2021 Jan 12;11:506. doi: 10.1038/s41598-020-79795-w (PMC7804315; doi:10.1038/s41598-020-79795-w)
Supplement: Supplementary file 1 — Supplementary Information. [file 41598_2020_79795_MOESM1_ESM.docx]

**COUETTE FLOW OF VISCOELASTIC DUSTY FLUID IN A ROTATING FRAME ALONG WITH THE HEAT TRANSFER**

Muhammad Bilal^1^, Salaha Khan^1^, Farhad Ali^*2,3^, Muhammad Arif^1^, Ilyas Khan^4^, Kottakkaran Sooppy Nisar^5^

^1^Department of Mathematics, City University of Science and Information Technology, Peshawar, Khyber Pakhtunkhwa, Pakistan.

^2^Computational Analysis Research Group, Ton Duc Thang University, Ho Chi Minh City, Vietnam.

^3^Faculty of Mathematics and Statistics, Ton Duc Thang University, Ho Chi Minh City, Vietnam.

^4^Department of Mathematics, College of Science Al-Zulfi, Majmaah University, Al-Majmaah 11952, Saudi Arabia.

^5^Department of Mathematics, College of Arts and Science, Wadi Al-Dawaser, 11991, Prince Sattam bin Abdulaziz University, Saudi Arabia.

*Correspondence and requests for materials should be addressed Dr Farhad Ali ([farhad.ali@tdtu.edu.vn](mailto:farhad.ali@tdtu.edu.vn))

**Appendix A-I**

$\rho C_{P}\frac{\partial T}{\partial t}=k\frac{\partial^{2}T}{\partial y^{2}}+4\alpha_{0}^{2}(T-T_{\infty}),$

By using dimensionless variables

$\begin{matrix} \theta=\frac{T-T_{\infty}}{T_{w}-T_{\infty}}, y^{*}=\frac{y}{d}, t^{*}=\frac{F_{0}t}{d}, \\ \rho C_{P}\frac{T_{w}-T_{\infty}}{\frac{d}{F_{0}}}\frac{\partial\theta}{\partial t}=k\frac{T_{w}-T_{\infty}}{d^{2}}\frac{\partial^{2}\theta}{\partial y^{2}}+4\alpha_{0}^{2}(T_{w}-T_{\infty})\theta, \\ \frac{\rho C_{P}F_{0}}{d}\frac{\partial\theta}{\partial t}=\frac{k}{d^{2}}\frac{\partial^{2}\theta}{\partial y^{2}}+4\alpha_{0}^{2}\theta, \end{matrix}$multiplying both sides by $\frac{d^{2}}{k}$

$$\begin{matrix} \frac{\rho C_{P}F_{0}d^{2}}{kd}\frac{\partial\theta}{\partial t}=\frac{\partial^{2}\theta}{\partial y^{2}}+\frac{4\alpha_{0}^{2}d^{2}}{k}\theta, \\ \frac{\rho C_{P}F_{0}d}{k}\frac{\partial\theta}{\partial t}=\frac{\partial^{2}\theta}{\partial y^{2}}+\frac{4\alpha_{0}^{2}d^{2}}{k}\theta, \\ Pe\frac{\partial\theta}{\partial t}=\frac{\partial^{2}\theta}{\partial y^{2}}+N^{2}\theta, \end{matrix}$$

Where:

$Pe=\frac{\rho C_{P}F_{0}d}{k}, N^{2}=\frac{4\alpha_{0}^{2}d^{2}}{k},$

$Pe=$Peclet number

$N^{2}=$Radiation parameter for optically thin fluid.

**Appendix A-II**

$q_{r}$is the radiative heat flux in $y-$direction which is defined as:

$q_{r}=-\frac{4\sigma*}{3k*}\frac{\partial T^{4}}{\partial y}$ A1

where $\sigma*$ and $k*$ represents Stefan-Boltzmann constant and mean absorption coefficient respectively. $T^{4}$can be expressed as a linear combination of the temperature by expanding $T^{4}$in a Taylor series about $T_{\infty}$and neglecting higher terms.

$T^{4}=T_{\infty}^{4}+4T_{\infty}^{3}(T-T_{\infty})+.........$ A2

$q_{r}=-\frac{4\sigma*}{3k*}\frac{\partial}{\partial y}\left(T_{\infty}^{4}+4T_{\infty}^{3}(T-T_{\infty})\right)$ A3

$q_{r}=-\frac{16\sigma*}{3k*}T_{\infty}^{3}\frac{\partial T}{\partial y}$ A4

Since over fluid is optically thin and as per Coogley. [50], $\frac{\partial q_{r}}{\partial y}$is approximated to be

$\frac{\partial q_{r}}{\partial y}=-4\alpha_{0}^{2}(T-T_{\infty})$ A5

$\alpha_{0}^{2}=\int_{0}^{\infty} k*\frac{\partial B_{\lambda}}{\partial T}d\lambda$ A6

$B_{\lambda}$is the Planks function.
